
JOURNAL INFORMATION
==============================
NLM Title Abbreviation: ScientificWorldJournal
Journal ID: ScientificWorldJournal
NLM Journal ID: 101131163
Journal ID: TSWJ

The Scientific World Journal

ISSN: 2356-6140
EISSN: 1537-744X
Publisher: Wiley

ARTICLE INFORMATION
==============================
PMCID: PMC6009385
PMID: 29973841
DOI: 10.1100/tsw.2002.64
Article ID: 989326
Article version: 1
Subjects: Short Report

The Four-State Equilibrium Unfolding of a scFv Antibody Fragment

Pedroso Idolka
Irún María P.
Machicado Claudia
Sancho Javier *
Departamento de Bioquímica y Biología Molecular y Celular. Facultad de Ciencias. Universidad de Zaragoza. 50009 Zaragoza, Spain
*Javier Sancho: jsancho@posta.unizar.es
Publication date: 2002
Electronic publication date: 2002 Jun 12
Volume: 2
First page: 136
Last page: 137
Copyright: Copyright © 2002 Idolka Pedroso et al.
Copyright year: 2002
License: This is an open access article distributed under the Creative Commons Attribution License, which permits unrestricted use, distribution, and reproduction in any medium, provided the original work is properly cited.
License URL: https://creativecommons.org/licenses/by/3.0/

==============================
Advances in Gene Technology: The Genome and Beyond –
Structural Biology for Medicine (Proceedings of the 2002 Miami
*Nature Biotechnology* Winter Symposium)
*TheScientificWorld* 2002, 2(S2), 136–137
ISSN 1532-2246; DOI 10.1100/TSW.2002.64

### THE FOUR-STATE EQUILIBRIUM UNFOLDING OF A scFv ANTIBODY FRAGMENT

Idolka Pedroso, María P. Irún, Claudia Machicado, and Javier Sancho*
Departamento de Bioquímica y Biología Molecular y Celular. Facultad de Ciencias. Universidad de Zaragoza. 50009 Zaragoza (Spain)
*jsancho@posta.unizar.es

**INTRODUCTION.** Single chain Fv fragments (scFv) are interesting alternatives to the use of whole antibody molecules[1]. We describe here an equilibrium intermediate of the thermal and urea unfolding that may compromise the stability of this class of molecules and constitute the main target for scFv stabilisation.

**METHOD.** The single-chain Fv antibody fragment against a hepatitis B surface antigen (anti-HBsAg scFv) was a generous gift from the Centro de Ingeniería Genética y Biotecnología, la Habana, Cuba. The equilibrium unfolding and spectroscopic characterisation of the scFv was performed essentially as described for other proteins studied in our laboratory[2].

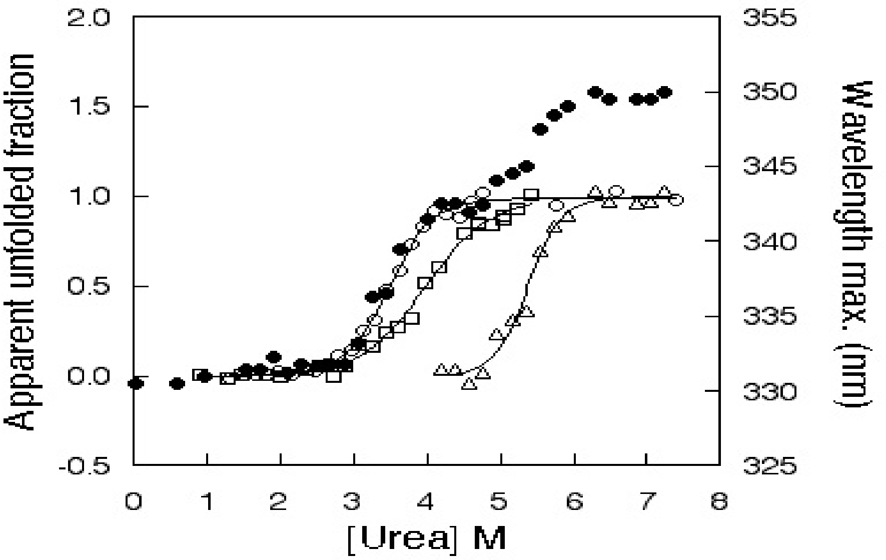

**FIGURE 1.** Urea denaturation of scFv at pH 7.0, followed by fluorescence intensity (o), far-UV CD (□) and wavelength of maximal fluorescence emission (•). The wavelength red-shift at higher urea concentration is additionally represented for better comparison (Δ).

**RESULTS.** At neutral pH and low protein concentration the scFv is a well-folded monomer and its urea and thermal denaturations are fully reversible. The noncoincidence of the fluorescence and circular dichroism transitions indicates the accumulation of an intermediate ($I_1$) not previously described in scFv molecules. In addition, at higher temperatures or higher urea concentrations, a red-shift in the fluorescence emission maximum reveals a second intermediate ($I_2$), similar to one already reported for other scFvs. The equilibrium unfolding of the anti-HBsAg scFv is thus four-state.

**DISCUSSION.** Our minimal model for both the urea and the thermal unfolding of the scFv molecule is thus:

**N<—> $I_1$<—> $I_2$<—>U**

In this model N, $I_2$ and U represent the species already characterised by the Plückthun group[3] while $I_1$ is a new intermediate. We have globally analysed the urea (and the temperature) unfolding data (as explained in [2]). The global analysis of the thermal unfolding suggests $I_1$ displays substantial secondary structure and some well-defined tertiary interactions. Its fluorescence properties are consistent with a disruption of the $V_L/V_H$ interface and a compact non-native conformation of the $V_L$ domain. The second intermediate probably contains a well-folded $V_H$ domain and a fully denatured $V_L$ domain, and unfolds at higher temperature in a noncooperative fashion. Global analysis of the urea unfolding data allows to calculate the N-$I_1$, $I_1$-$I_2$, and $I_2$-D free energy differences. Although the N-D free energy difference is very large, the N-$I_1$ one, representing the 'relevant' conformational stability of the scFv, is small. Stabilising the native state relative to the $I_1$ state thus requires an improvement to the ´relevant´ conformational stability of this scFv molecule.

### REFERENCES

1. Plückthun, A. (1994) In Immunochemistry. Van Oss, C.J. and van Regenmortel, M.H.V., Eds. Marcel Dekker, New York. pp 201–235.
2. Irún, M.P., Garcia-Mira, M.M., Sanchez-Ruiz, J.M., and Sancho, J. (2001) J. Mol. Biol. 306, 877–888.
3. Wörn, A. and Plückthun, A. (1999) Biochemistry 38, 8739–8750.
